# Saliva soluble HLA as a potential marker of response to interferon-β1a in multiple sclerosis: A preliminary study

**DOI:** 10.1186/1742-2094-4-16

**Published:** 2007-07-01

**Authors:** Alireza Minagar, Irena Adamashvili, Roger E Kelley, Eduardo Gonzalez-Toledo, Jerry McLarty, Stacy J Smith

**Affiliations:** 1 Department of Neurology, LSU Health Sciences Center, Shreveport, Louisiana, USA; 2 Department of Radiology, LSU Health Sciences Center, Shreveport, Louisiana, USA; 3 Department of Internal Medicine, LSU Health Sciences Center, Shreveport, Louisiana, USA

## Abstract

**Objective:**

Potential surrogate markers of disease activity, including response to therapy, are particularly important in a neurological disorder such as multiple sclerosis (MS) which often has a fluctuating course. Based upon previous studies in our laboratory, we hypothesized that measurement of soluble HLA (sHLA) molecules class II in saliva of MS patients can serve as marker of therapeutic response to high dose interferon beta-1a.

**Methods:**

We measured the expression patterns of sHLA-II in saliva in 17 patients with relapsing/remitting MS and compared the results to clinical course and brain MRI. For comparison purposes we also assayed the saliva sHLA-II levels in 53 normal control subjects. Solid phase ELISA was used for measurement of sHLA-I and sHLA-II concentrations at baseline and after three and six months of treatment with high dose interferon beta-1a (IFN β-1a).

**Results:**

The mean saliva sHLA-ll levels in MS patients was significantly higher than normal controls (354 ± 42 unit/mL vs. 222 ± 18 unit/mL, t= 8.16, p < 0.003). Comparison of saliva sHLA-II values before and after treatment with IFN β-1a revealed a consistent increase in mean concentration. The increase in saliva sHLA-II values (354 ± 42 unit/mL at baseline versus 821 ± 86 unit/mL at 3 months and 776 ± 63 unit/mL at 6 months, in unit/mL, p < 0.001 for both comparisons) was associated with a stable clinical course and a decline of the number of contrast-enhancing lesions on brain MRI. Comparison of the volume of T2-weighted lesions and the number of black holes on T1-weighted images did not reveal any significant changes (during pre-treatment versus post-treatment month 6) or any correlations with saliva sHLA-II levels. Saliva sHLA-I levels were not detectable.

**Conclusion:**

Serial measurement of saliva sHLA-II may serve as a potential marker of therapeutic response to IFN β-1a. Larger clinical studies involving more RRMS patients over longer periods of time are needed to further test the significance and value of saliva sHLA-II as an accurate marker of therapeutic response to beta-interferons.

## Background

The Human Major Histocompatibility Antigens (HLA) are generally cell bound, but trace amounts exist in soluble forms which circulate in serum, plasma, and other human body fluids [1]. These soluble HLA class-I (sHLA-I) and class-II (sHLA-II) molecules may have immunomodulatory function [2-4]. Normal individuals have stable concentrations of sHLA-I and sHLA-II in their serum [1]. However, the serum level of sHLA-I is significantly elevated in patients with various inflammatory diseases [5-8] although this is not necessarily the case for serum sHLA-II levels [9,10]. Preliminary evidence suggests that patients with systemic lupus erythematosus (SLE) are at increased risk of developing active disease in the presence of high sHLA-I levels in the saliva, while sHLA-II level has not been observed to be elevated in rheumatological diseases [11]. Typically, sHLA-I exists in very low quantities in the saliva, sweat, urine and/or tears of normal individuals, while sHLA-II is routinely detectable in all these body fluids [1].

The potential role of soluble HLA in the pathogenesis of multiple sclerosis (MS) is not presently established. Alterations in sHLA-I and sHLA-II levels in the serum and CSF of MS patients has been reported [12-14]. A trend toward increased production of sHLA-I in the serum and CSF was observed in MS patients after immunomodulatory therapy [15]. On the other hand, sHLA-II in the serum of untreated MS patients was found to be significantly elevated compared to values obtained from MS patients receiving corticosteroid treatment. [12] Hypothetically, one would expect the measurement of sHLA in CSF would be most likely to reflect CNS disease activity. Measurement of such a biological marker of disease activity could potentially serve as a monitor of response to immunomodulatory treatment in MS. However, a biological marker of disease activity, as well as response to therapy, must be reliable, noninvasive, and preferably of reasonable cost in order to be performed in a serial fashion reflective of the natural history of the relapsing/remitting form of (RRMS).

We have previously reported a correlation between CSF and saliva sHLA-II levels in MS patients [16]. We have now extended our investigation to assess the possible response of saliva sHLA-II levels in RRMS patients at baseline and following immunomodulatory therapy with interferon-beta1a (Rebif) (IFN-β1a).

## Methods

The study was approved by Institutional Review Board of Louisiana State University Health Sciences Center in Shreveport and signed informed consent was obtained from all participants.

### Population studied

Saliva specimens from 17 consecutive Caucasian patients with RRMS, defined by the McDonald criteria [17], were collected and analyzed. None of the patients were on immunomodulating therapy or immunosuppressive therapies for at least six months prior to entrance into the study. Two patients had experienced two clinical relapses during the six months prior to study entry, while the others had only one relapse prior to study initiation. Because there is a high degree of racial variation in the gene frequencies of HLA, we limited study participation to Caucasians born in the United States and residing in Louisiana.

### Collection of samples

Each subject involved in the study was asked to expectorate saliva into a test tube preceded by rinsing of the mouth with sterile water as previously described [16]. In addition, saliva specimens from 53 healthy age and sex matched individuals were used for comparison. Collected saliva samples were stored at -20C until subsequent assay.

### Patient monitoring

After obtaining the first saliva specimen, patients were treated with IFNβ-1a (Rebif^®^) 44 mcg subcutaneously three times weekly. All patients underwent neurological examination at baseline as well as at three and six months and this included expanded disability status scores (EDSS) during each visit.

### MRI protocol

Brain MRI was performed using a 1.5 T machine with a standard quadrature head coil. The imaging protocol included sagittal T1-, axial T1-, T2-weighted, and fluid attenuated inversion recovery (FLAIR) images. All MRI scans were performed before and after (Gd-DTPA) infusion. Axial T2-weighted and pre- and post-contrast T1-weighted images were used for assessment of MS plaques. Comparisons were made between all 17 MS patients who were sub-grouped into either those with and those without enhancing lesions on their MRI scans. MR imaging was performed prior to and after six months of treatment with IFN β-1a. Volumetric analysis of T2-weighted lesions was performed by a neuroradiologist who delineated the T2-weighted lesion as the area of interest; the volume was calculated by multiplying the total area of interest by the slice thickness. The results were expressed in cubic milliliters (Zivadinov 2001). The numbers of T1-weighted black holes, pre- and post-treatment, were determined qualitatively by number count.

### Measurement of soluble HLA

A solid phase ELISA was used to quantitate s-HLA-I and s-HLA-II in the saliva obtained from study subjects. The immuno-affinity purification and quantitation of s-HLA-I and s-HLA-II has been previously described [18]. This assay is highly reproducible and not influenced by anticoagulants, freezing or thawing. Briefly, test specimens were added to appropriate wells containing an anti-Class I (w6/32) or anti-Class II (Ab 2.06) monoclonal antibody (Mab) coated beads. The reaction proceeds 30 minutes for sHLA-I and 2 hours for sHLA-II at 45°C. The beads are then washed three times with distilled water. Following this, 200 ul of peroxidase labeled anti-beta2 microglobulin (L368) for sHLA-I and L.2.03 Mab for sHLA-II were added to each bead and incubated for an additional hour at 45°C. After additional washes, the color reaction was started by adding O-phenylenediamine as a substrate. The color intensity is proportional to sHLA concentration. Absorbance was measured at 492 nm. Saliva levels of sHLA-II were measured at baseline as well as at 3 and 6 months after treatment with IFN β-1a. Saliva sHLA-II concentrations pre and post-treatment were compared to the neurological status of the patient as well as to the MRI brain scan findings.

### Statistical analysis

Using the Friedman non-parametric method [19], we compared the mean values for saliva sHLA-II in study subjects and normal controls. Paired comparison of changes from baseline to post-treatment was performed with use of Wilcoxon signed rank test. Correlations were determined with Spearman's non-parametric correlation method.

## Results

**Figure 1:**
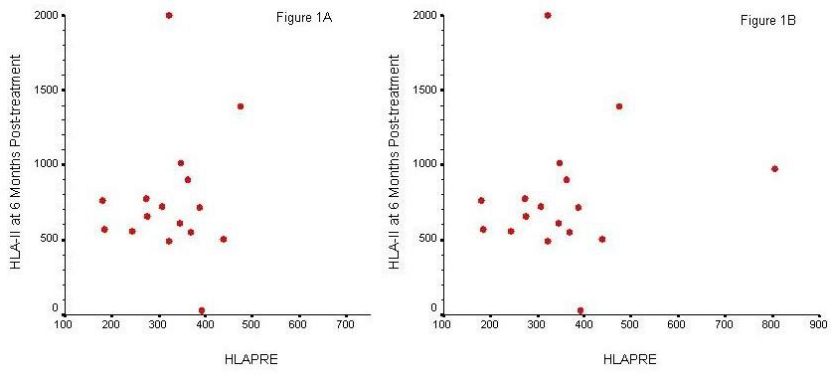
(A) Scattergram of baseline and three-month levels of sHLA-II. The correlation is significant, ρ = .51, p = 0.035. (B) Scattergram of baseline and six-month levels of sHLA-II. The correlation is not significant, ρ = .063, p = 0.81.

Demographics of study subjects are presented in Table 1. *Soluble HLA saliva *levels. Scatter plots of distribution of saliva levels of sHLA-II of MS patients pre- and post-treatment with IFN-β1a is presented in two scatter plots (Figures 1A and 1B). All study subjects with RRMS had measurable amounts of sHLA-II in their saliva and in all subjects increases in saliva sHLA-II levels following treatment with IFN-β1a (at month 6 post-treatment) were detected. The mean value of sHLA-II was 354 ± 42 (unit/mL) baseline, 821 ± 86 (unit/mL) at month 3, and 776 ± 63 (unit/mL) at month 6 (p < 0.001) for both. Correlation analysis of pre-versus post-treatment saliva sHLA-II levels at month 3 was significant r =.51, p=.035; however, was not significant for measurement at month 6 r =.063, p = .81. Furthermore, we did not observe any discernible relationship between pre- and post-treatment saliva sHLA-II levels and subjects' clinical status. Additionally, we did not observe any specific relationship between the baseline saliva sHLA-II levels and follow up values. All normal controls had detectable amounts of sHLA-II in the saliva with a mean value of 222 ± 18 unit/mL.

**Table 1:** Demographic and MRI features of the MS subjects

Number of MS patients	17 (F/M = 11/6)
Age (Mean ± SD)	29 ± 3 years
MS duration prior to diagnosis	9 ± 2 months
Number of T1-weighted post-contrast enhancing lesions	
At least one at month 0	(N = 6)
At least one at month 6	(N = 0)
Number of relapses during the six months prior to study entry	
Two relapses	(N = 2)
One relapse	(N = 15)

### Neurological status

During the six months of treatment, only one patient had a documented clinical relapse with optic neuritis.

### Correlation with MRI brain scan results

Of particular interest, the increase in sHLA-II values was associated with a decline on brain MRI activity as demonstrated by post-contrast T1-weighted axial brain images. Initially, six patients had contrast enhancing lesions on axial post-contrast T1-weighted brain MR images. Comparison of brain MRI at months 0 and 6 did not show any new contrast-enhancing lesions. Comparison of the mean volumes of T2-weighted lesions as well as the average count of T1-weighted black holes, pre-versus post-treatment, did not show any significant differences (p = 0.381 and p = 0.89 respectively). Additionally, we did not observe a correlation between the volume of T2-weighted lesions or the number of T1-weighted black holes with saliva sHLA-II levels.

### Correlation of sHLA levels with disability status

At six months, all subjects exhibited a one-grade or greater decrease in their EDSS scores (p < 0.001) which occurred in association with elevated levels of saliva sHLA-II levels at month 6.

## Discussion

There are a number of important reasons to have a reliable surrogate marker for disease activity in RRMS. As the name implies, there can be a significant day-to-day fluctuation in this form of MS. Furthermore, response to interferon beta, the most common form of immunomodulating therapy, can vary with only 30 to 40% of patients reported to have a good response [20,21]. This may, in part, be related to the formation of anti-interferon beta neutralizing antibodies [22]. In an effort to address this issue, in terms of a marker of response, levels of interferon inhibitory activity (IIa) were measured in a recent study and reported to be an indicator of therapeutic efficacy [23]. Efforts have also been made to determine clinical and demographic indicators of disease activity in an effort to predict the clinical course over time [24]. Colucci et al. [25] measured CSF levels of protein 14-3-3 in patients with demyelinating disorder and reported that, in some patients, protein 14-3-3 may serve as a marker of disease severity and risk to develop disability. The monitoring of MS patients' response to therapy, with a surrogate marker, may have additional value as particularly effective immunomodulatory activity, such as that observed with natalizumab, may have a potential downside [26] that is better to detect sooner rather than later.

The mechanisms for secretion or excretion of sHLA class I and II in various human body fluids remain unknown. It has been suggested that liver cells and activated immunocompetent cells actively secrete sHLA-I into the serum [27]. sHLA-II may also be shed or secreted into serum and other body fluids by certain immune processes. However, the potential immunoregulatory function of these molecules can not be elucidated as currently there are no specific monoclonal antibodies for class II allotypes available. Furthermore, it would be unlikely that the same mechanism(s) would account for sHLA release in various body fluids as immune reactive cells are not typically present in body fluids, except in pathological states.

We demonstrated, in studies of sHLA-I in serum, sweat, and saliva, that saliva contains polymorphic structures identical to those of serum sHLA-I, but the concentration of sHLA-I was very small [1]. It is possible that HLA appears in other body fluids en route to sites of catabolism or excretion as a consequence of a specific physiological event. Serum sHLA-II has been reported to be low or undetectable in most normal individuals tested [9], while it is commonly present in the saliva, sweat and tears of normals [1,11]. Soluble HLA-II has also been reported in the synovial fluid of patients with active rheumatoid arthritis, but not in the serum [10,28], which in turn supports the concept of selective distribution of sHLA-II within body fluids.

Based on the literature to date, sHLA levels appear to correlate with RRMS disease activity [12-16]. However, our knowledge about the dynamics of soluble HLA in MS patients, as an indicator of both disease activity and response to immunomodulatory therapy, is marginal and further studies are needed. Recently, Fainardi et al [15] described a trend towards increased production of sHLA-I in the serum of patients with MS following the treatment with IFN β-1b. However, we have observed a decrease in serum sHLA-II levels after therapy with IFN β-1a in MS patients [29]. A recent report by Mitsdoerffer et al [30] suggests that although both IFN-β and IFN-γ significantly increase sHLA-G1 and sHLA-G5 (non-classical HLA-class-I) expression by monocytes *in vitro*, IFN-β treatment is associated with higher upregulation of HLA-G compared to classic HLA-I molecules than stimulation with IFN-γ. Since monocyte-derived HLA-G inhibits autologous CD4 T cell activation, its upregulation by IFN-β was considered as one of the mechanisms of action of IFN-β.

In the present study, longitudinal measurement of saliva sHLA-II levels over a six month period demonstrated elevation of saliva sHLA-II levels in association with IFN β-1a therapy in MS (p < 0.0001). Collectively taken, our results indicate that treatment of MS patients with IFN β-1a produces a rapid, consistent and persistent elevation of saliva sHLA-II after six month of therapy. Such a pronounced response may indicate that IFN β-1a up-regulates the expression of HLA class II genes/molecules. The elevation of saliva sHLA-II in our patients, in association with clinical and MRI stability, may also be indicative of a favorable therapeutic response to IFN β-1a. Further studies involving larger cohorts of MS patients and over longer follow up periods are necessary to assess the potential of sHLA-II as a marker of disease activity and response to therapy in MS.

## Competing interests

Dr. Minagar has received an independent medical grant from Serono, Inc.

## Authors' contributions

AM and IA designed the clinical research protocol, provided expertise for laboratory measurement of saliva soluble HLA-II, and prepared the manuscript.

REK and EGT contributed to this manuscript by recruiting MS patients, interpreting the neuro-radiology data and preparing the manuscript.

JM has contributed to this manuscript by doing statistical analysis and generating the figures.

SJS has contributed to this manuscript by recruiting MS patients, collecting the data, preparing the saliva specimens and preparing the manuscript.

All authors have read and approved the contents of the final mansucript.
